# Anaemia, anthropometric status and associated factors among electronic waste recyclers, women and children residents of Agbogbloshie, Ghana

**DOI:** 10.21203/rs.3.rs-6922246/v1

**Published:** 2025-07-15

**Authors:** Sylvia A. Takyi, Jaclyn M. Goodrich, Thomas Robins

**Affiliations:** University of Michigan; University of Michigan; University of Michigan

## Abstract

**Background:**

Anemia is a significant public health issue, particularly in communities exposed to environmental hazards and poor nutritional conditions. Limited data is available in Ghana regarding the health and nutritional status of vulnerable groups like women and children residing in polluted areas such as Agbogbloshie in Accra.

**Aim/ Objective:**

We assessed the prevalence of anemia and its association with sociodemographic and anthropometric factors among e-waste recyclers, women, and children living in Agbogbloshie, a community known for its e-waste recycling activities.

**Methods:**

A cross-sectional study was conducted among male e-waste recyclers, women of reproductive age, and children aged 5–17 years at Agbogbloshie, Ghana in 2022. Data on sociodemographic and socioeconomic factors were collected using a semi-structured questionnaire. Anthropometric measures were conducted, whilst onsite hemoglobin levels were measured using the HemoCue 301+ machine. Among children, the weight-for-age (WAZ) score and length-for-age (LAZ), BMI-for-age and Mid-Upper Arm Circumference (MUAC)-for-age scores were measured based on the WHO growth charts and the WHO anthropometry calculator. Linear regression models were used to identify sociodemographic factors, anthropometric measures and other factors associated with anemia.

**Results:**

Anemia was prevalent among women and children in this study. Furthermore, anemia prevalence was higher among female adults (54.90%) compared to the male recyclers (9%) [χ^2^ = 38.47, p < 0.001]. Some (45.56%) children between the ages of 12 to 17 years did not meet the recommended WHO reference for hemoglobin levels. Sociodemographic factors, such as lower educational level, gender, age and socioeconomic status were significantly associated with anemia. Higher BMI was positively associated with higher hemoglobin levels in e-waste recyclers (*β* = 0.098; 95% Cl: 0.004, 0.193; *p* = 0.04). However, we did not find an association between anthropometric measures (height-for-age, weight-for-age and BMI-for-age) and hemoglobin levels of children in this study (p > 0.05). Nonetheless, children who consumed food from less than five food groups had significant reduction in hemoglobin levels [*β* = −1.789; 95% Cl: −3.531, −0.047; *p* = 0.04].

**Conclusion:**

Anemia remains a major public health concern in Agbogbloshie, with significant associations between anemia and sociodemographic factors and some anthropometric measures. The high prevalence of anemia in women and children highlights the need for targeted interventions addressing nutritional deficiencies, environmental exposures, and socioeconomic disparities.

## Background

Despite efforts by the Ghanaian government to put an end to informal e-waste recycling activities at Agbogbloshie, one of the world’s most notorious e-waste recycling hubs, these harmful practices continue to persist. While several epidemiological studies have been conducted on the e-waste recycling among males, none has focused specifically on the impact on women and children. This is concerning, as exposure to the toxic substances like lead (Pb), cadmium (Cd), arsenic (As) and mercury (Hg) associated with informal e-waste recycling can have devastating effects on the health of children living in the polluted area; including stunted growth and neurodevelopment, respiratory and cardiovascular issues, endocrine disruption, genetic abnormalities, and haematological problems [1, 2, 3, 4, 5].

Anemia is a complex hematological condition with multiple determinants. Among the most widely reported determinants are demographic factors, such as age, gender, and ethnicity[6], as well as geographical location [7], physical activity levels [8], and nutritional status [9]. This medical condition is a major cause of illness and death, affecting about 1.62 billion people globally. Among women of reproductive age, the prevalence rate is 57.1%, with the highest rates found in Central (61%) and West Africa (61%)[10]. According to the World Health Organization’s (WHO) classification, anaemia is a severe public health issue. In Ghana, more than 65% of children and 42% of women are classified as anaemic [11]. Early detection of anemia may lead to its rapid amelioration. While blood Hb estimation is not a feasible option to predict the occurrence of anemia in the entire resource-poor group living in a highly polluted area like Agbogbloshie, anthropometric parameters such as body mass index (BMI), height, weight, waist circumference (WC), waist-to-hip ratio (WHR), and waist-to-height ratio (WHtR) have been found to hold close associations [12, 13, 14, 15].

Tragically, many children still live, attend school, and play in close proximity to e-waste recycling centres, where they are at risk of exposure to harmful substances such as Pb and Hg. Due to their smaller size, less developed organs, and accelerated rate of growth and development, children are particularly vulnerable to the toxic pollutants associated with e-waste. They absorb more chemicals in proportion to their size and have a lower ability to digest and eliminate hazardous chemicals from their systems [16]. Exposure to e-waste has been discovered to have a substantial impact on the prevalence of anaemia among residents. This is primarily due to the presence of toxic chemicals, such as Pb and Hg [17, 18, 19, 20], which are often found in e-waste. These chemicals can have a detrimental effect on the body’s ability to produce red blood cells. For example, Wang et al. found that Pb exposure significantly inhibits Hb synthesis in children living in e-waste dismantling areas compared to those residing in non-e-waste dismantling areas [21].

Given the increasing prevalence of anemia that can have severe consequences if left untreated among vulnerable groups, such as women and children living in polluted resource-poor communities, it is crucial to investigate critical clinical conditions such as anemia and anthropometric measures in these communities. By measuring anaemia prevalence and anthropometric indices, we hope to provide valuable baseline insights into the impact of pollution on the health of not only e-waste recyclers, but women and young children living in such polluted areas. This information can be used to develop targeted interventions and policies to improve the health and well-being of individuals living in polluted areas. We recognize the importance of addressing the issue of pollution and its impact on human health. Our study is a key step towards understanding the challenges faced by individuals living in polluted areas and developing effective solutions to mitigate the negative effects of pollution.

## Materials and Methods

### Study Design

We employed a cross-sectional design during the month of December 2022 to measure the prevalence of Anaemia and anthropometric indices among male e-waste recyclers, women and children resident at Agbogbloshie. Data collection was conducted in December 2022 to coincide with the harmattan season, a period characterized by distinct environmental conditions, including northeasterly winds, lower temperatures, and reduced humidity. These factors significantly influence the dispersion of airborne pollutants, including toxic metals released during e-waste activities. Collecting data during this season allowed the study to assess potential peak exposure levels among vulnerable populations, such as women and children in nearby communities. The study was approved by the Ethical & Protocol Review Committee of the College of Health Sciences, University of Ghana.

### Study area

Agbogbloshie, a community located in central Accra, has gained worldwide notoriety for its informal e-waste recycling practices. However, this bustling community is more than just a recycling hub. It is home to one of the largest food markets in Ghana and situated on the banks of the Odaw River and the Korle-Lagoon, covering an area of 1.46 km^2^ and housing an estimated population of 80,000 people [22, 23, 24]. The informal community, also known as “Old Fadama,” lies to the southwest of the recycling area and is home to most of the recyclers, traders, street hawkers, head potters, children and families. Majority of the residents are culturally Dagombas or Konkombas who migrated from the northern part of Ghana in search of better opportunities. The Agbogbloshie scrap yard, located near informal community residences, is notorious for emitting heavy clouds of smoke from the daily burning of e-waste materials. This practice is used to recover valuable metals, such as copper, as workers–who are paid very limited wages–rely on burning as the quickest and most accessible method to extract these materials, despite its harmful consequences for all involved [25, 26, 27].

### Population, Sampling Method, Eligibility Criteria, and Recruitment

This study, which followed the West Africa GEOHealth II Project, aimed to investigate the health risks associated with e-waste recycling in Agbogbloshie, Accra. Over the course of one month, a total of 206 participants were recruited, including e-waste recyclers (burners, dismantlers, and collectors/sorters), women, and children at Agbogbloshie. E-waste recyclers made up 50% of the sample size, with women and children each constituting approximately 25%. Windspeed and direction significantly influence exposure levels among e-waste recyclers, women and children, determining who faces higher exposure at any given time and over extended periods, such as days or months. During the harmattan, wind speeds and directions can fluctuate [28], increasing the risk for e-waste workers who may unknowingly position themselves downwind of burning sites, where pollutant concentrations are higher. It is important to note that e-waste recyclers are at a higher risk of exposure due to tasks such as open-air burning. Therefore, it is crucial to recruit more of this group to better understand the differences in exposures per recycler-task performed. By doing so, we can gain a more comprehensive understanding of the health risks associated with e-waste recycling and develop effective interventions to protect the health of those involved

To ensure that eligible participants were informed and familiarized with the objectives and procedures of the study, a community ‘gong-gong’ was sounded, and participants were given a thorough explanation of the study. The study was open to male adult e-waste recyclers who were 18 years and above and had worked at the e-waste site for at least six months. Children aged 5 to 17 years were also given the opportunity to participate with the consent of a parent or guardian. Adult women of reproductive age were also enrolled in the study, however but we ensured that only women who were not currently menstruating were eligible to participate. This was done to control for the potential impact of blood loss on the study results. Prior to the start of the study, informed consent was obtained from all adult participant, while children provided assent with written consent from their parents. However, individuals with mental or physical disabilities that may interfere with their ability to understand the informed consent or complete metabolic health status measures were not eligible to participate.

### Data Collection

#### Anthropometric Measures

Trained research assistants conducted anthropometric measurements using calibrated equipment to measure weight, height, Mid-Upper Arm Circumference (MUAC), and waist-to-hip ratio. These measurements were taken with precision and accuracy to ensure reliable data collection.

Participants’ height of both adults and children was accurately measured and adjusted to the nearest 0.1 cm using a Seca Stadiometer (Seca, Germany). To ensure accuracy, the participants were asked to stand upright on a flat surface without shoes, with their heels and occiput (back of the head) pressed firmly against the stadiometer. Weight was also measured and recorded to the nearest 0.1 kg using a portable Seca Scale (Seca770; Hamburg, Germany). BMI z-score, weight and height for age z-score were calculated from these parameters [29]. Furthermore, the data was standardized by age and according to the World Health Organization (WHO) growth standards to calculate BMI-for-age z-scores for children between the ages of 5 and 17 years [30]. These scores were then used to categorize the children’s sample population into six weight categories: Severe thinness: <−3SD, Thinness: <−2SD, healthy-weight (−2 ≤ z ≤1), at-risk-of-overweight (1 < z ≤ 2), overweight (2 < z ≤ 3), and obese (z > 3), based on WHO growth standards [30, 31]. However, BMI for adults were categorized as: underweight: >18.5kg/m^2^; normal weight: 18.5 to 24.9 kg/m^2^; overweight: 25–29.9 kg/m^2^ and obese: 30 kg/m^2^ or more. In order to determine the waist to hip ratio to assess adiposity, we measured the circumference of the waist (WC) and the hip (HC) using an inextensible anthropometric with each participant standing upright with their arms at their sides and feet together during the measurement process [12, 32]. The Mid-Upper Arm Circumference (MUAC) was measured among children using a standard UNICEF MUAC tape. Specifically, the left arm circumference was measured in centimeters at the midpoint between the acromion and shoulder. All anthropometric measurements were taken twice and the average of the two measurements was recorded once a difference is observed.

#### Questionnaire:

Using structured questionnaires adopted from the GEOHealth-II longitudinal cohort study [26, 33, 34], baseline interviews were conducted among recyclers, women, and children to assess their demographics, past and current potentially adverse exposures, and personal and family medical history. The interviews were conducted in English, Dagbani, or Hausa, depending on the participants’ preferred language. The focus of the interviews was on environmental tobacco/exposure to environmental tobacco smoke, exposure to indoor cooking using biomass fuels, type of housing, detailed job history, interference with the ability to work or with other activities of daily living, medication use, and health services utilization. The purpose of these interviews was to gather important information about the participants’ health and living conditions. Specifically, we wanted to understand the potential health risks associated with their work and living environments. By conducting these interviews in the participants’ preferred languages, we were able to ensure that they felt comfortable and understood the questions being asked. During the interviews, we asked detailed questions about the participant’s medical history, including any diagnosed metabolic illnesses like diabetes, hypertension, dyslipidemia, obesity, and other reported symptoms. We also asked about any interference with their ability to work or with other activities of daily living, medication use, and health services utilization.

#### Biological Sample Collection

In the field, non-fasting venous blood of each participant was collected and analyzed by a trained phlebotomist for hemoglobin (Hb) concentration using a HemoCue instrument (HemoCue 301, Angelholm, Sweden) [35]. The HemoCue is a portable, user-friendly, and factory-calibrated device ideal for field work as it only requires a capillary finger-prick or venous sample collection. Our collection techniques followed globally endorsed standards [36, 37, 38]. The HemoCue Hb301 system accurately determines the concentration of hemoglobin by measuring the absorbance of whole blood at an Hb/HbO_2_ isosbestic point [35]. This device employs a double wavelength measuring method, utilizing 506 nm and 880 nm, to compensate for turbidity. The results are displayed numerically in g/dL at the time of the measurement, providing precise and reliable data. In the current study, we recorded Hb results displayed on the HemoCue by manually entering them into the STATA analytical software for statistical analysis. Dual entries were done to avoid data entry errors.

#### Diagnostic criteria for Anemia

Anemia prevalence was calculated with use of Hb concentration cut-offs defined by the WHO thresholds for children (males and females) (Hb < 11.0 g/dL), non-pregnant women (Hb < 120 g/dL) and adult males (Hb < 13g/ dL)[39, 40]. Using the WHO definition according to age classifications, Hb levels < 11.5 g/dL in children (both males and females) aged 6–11 years were considered anemic. Meanwhile, adolescents between the ages of 12 to 14 years, both male and female, were considered anemic if their hemoglobin level was < 12g/dL. Males aged 15 years and above, were considered anemic with Hb of < 13 g/dL, while for females aged 15 years were considered anemic with Hb of < 12 g/dL. For classification into mild, moderate and severe anemia per age and gender of participant, Hb levels were categorized based on guidelines published by in the Facilitator’s Guide to Training Health Workers in Ghana to Measure Hemoglobin and Assess Anaemia with the HemoCue^®^ Hb 301 Device [37]. Using WHO recommended categories for anemia severity [37], see Appendix A.

### Data Analysis

#### Anthropometric Analysis:

In children, weight-for-age (WAZ) score and height/ length-for-age (LAZ) and MUAC-for-age scores using the WHO growth charts and the WHO anthropometry calculator [30, 41, 42, 43, 44]. These were calculated as key indicators to identify signs of malnutrition among children within the 5-to-17-year age range. For instance, the MUAC-for-age Z score has been found to be equally effective as the body mass index-for-age Z score in assessing health risks associated with undernutrition among African school-aged children and adolescents [45, 46].

#### Dietary Diversity Scores (DDS):

Following the recommendations by the Fanta (Food and Nutrition Technical Assistance) Project which focuses on improving dietary diversity and nutrition outcomes worldwide, particularly in low-resource settings [47], we calculated a dietary diversity score for the male e-waste recyclers, women, and children. Data on food consumption was collected using a 48-hour dietary recall. The score was based on 9 food groups, including cereals, dairy, meat, fruits, and vegetables. People who consumed food from fewer than 5 groups were categorized to have low dietary diversity, while those who ate from 5 or more groups were classified to have high dietary diversity.

### Statistical Analysis

The data were stratified by age and sex of participants. Continuous variables, such as age, Hb concentrations, and anthropometric measures, were analyzed using means, standard deviations, medians, and interquartile ranges. ANOVA was used to determine the difference between mean Hb levels measured among participants by key categorical variables. Binary variables, such as anemia status, smoking, and alcohol intake were compared using Pearson chi-square tests. To identify demographic and anthropometric measures that predicted anemia among participants, ordinary least square regression models were utilized. All analyses were conducted using Stata (StataCorp, College Station, TX, USA).

## Results

### Sociodemographic and anthropometric characteristics of the study participants

#### Socio-demographics characteristics

##### Children (male and females under 18 years)

The mean (± SD) age of children was 13.27 (± 2.77) years, ranged between 6 to 17 years. Over half of the children (62.50%) were at least enrolled in primary school (Appendix B). The majority of the children were male, and many of them had fathers who worked at the e-waste recycling site in Agbogbloshie. These children often visited their fathers during work hours. The male children spent more time (6.18 ± 4.65 hours) at the e-waste site, compared to their female counterparts (3.35 ± 4.74 hours).

##### E-waste recyclers and adult women

The mean age of recyclers and women was 26.08 (± 8.19) ranging, from 18 to 55 years (Appendix B). The e-waste recyclers (26.98 ± 8.21years) had a slightly higher average age of compared to the women (24.35 ± 8.19) (*p* = 0.009). More than half of the adults (55.07%) earned between 21 to 100 GH cedis (~$1.8–8.8 USD), a day; with most of these adults being the e-waste recyclers. Additionally, approximately half of the adults were married. Most of the e-waste recyclers than adult women had at least primary or junior high school education (see Appendix B). Meanwhile, the women in this study mostly engaged in informal trading activities including cooked or fresh food products etc.

##### Anthropometric Measures

We analyzed the age-wise comparison of height for age z-score (HAZ) in children aged between 5 to 17 years in this study. Average BMI-for-age z-score measured among children aged 5 to 17 years was 0.47 (± 1.94) kg/m^2^. While the majority of children (54.17%) were of healthy weight, approximately 16% were overweight. A higher proportion of girls compared to boys were either overweight or obese (Appendix C). Conversely, more boys than girls experienced wasting in this study. Furthermore, the mean height-for-age (stunting) z-score was 29.60 (± 4.51) cm, while the mean weight-for-age z-score was 20.66 (± 6.51) kg among the children. Nonetheless, significant differences were found between male and female height/length for age measured among children (z = 3.032; *p* = 0.002). The mean Mid-Upper Arm Circumference (MUAC) for children was 16.97 (± 2.56). However, half (50%) of the children assessed were found to have severe acute malnutrition, with a higher prevalence among males. Height-for-age measured was mostly normal among both males and females (Table 1).

The recyclers (66.56 ± 11.57) kg were found to have significantly higher weight than the women (61.84 ± 13.17) kg who participated in the study (z = 2.32; *p* = 0.02). Among the e-waste recyclers and women, mean BMI measured was 23.63 (± 6.27 kg/m^2^). However, the mean BMI of adult women (24.82 ± 8.95 kg/m^2^) was slightly higher compared to the e-waste recyclers (23.02 ± 4.20 kg/m^2^) (Table 1).

##### Dietary Diversity of E-waste recyclers, Women and Children

Generally, mean (SD) dietary diversity score was 3.70 (±1.7) and the minimum score was 1 out of 9 and the maximum score was 6 out of 9. Minimum dietary diversity, defined as consumption of foods from five or more food groups, was practiced by 33 (16.26%) of 206 study participants. The common food group consumed were cereals and cereal products (97.55%) followed by fruits and vegetables (95.83%), meat and fish (78.64%) and then legumes, nuts and seeds (66.50%) (Table 2). Iron containing foods such as dark green leafy vegetables and organ meat rich in iron were poorly consumed among the e-waste recyclers, women and children. Not all women (61.11%) and children (72.92%) consumed meat and fish products. Some recyclers (57.69%), women (62.96%) and children (52.09%) consumed other vitamin A-rich fruits and vegetables. Eggs and milk and dairy products were poorly consumed among the e-waste recyclers, women and children.

##### Prevalence of Anemia among E-waste recyclers, Women and Children

The mean Hb level measured among children was 11.68 (± 2.66)g/dL. Although not statistically significant, the lowest level of Hb was measured among female children (10.40 ± 4.65 g/dL) aged between 15–17 years (Table 3). Furthermore, Hb levels measured among most (45.65%) children aged between 12 to 17 years were below their recommended reference. Average Hb levels measured among women and e-waste recyclers was 13.58 ± 2.20g/dL. The mean levels of Hb were significantly higher among the e-waste recyclers (14.65 ± 1.25g/dL) compared to adult women (11.60 ± 2.30 g/dL) at Agbogbloshie (*p* < 0.001). Among children, anemia was more prevalent among males than females (Table 4). Furthermore, anemia prevalence was higher among female adults (54.90%) compared to the recyclers (9%) (χ^2^ = 38.47, p < 0.001) (Table 4).

##### Correlation between Hemoglobin level, BMI, MUAC and waist to hip ratio of e-waste recyclers, women and children (N = 206)

Overall, a weak positive correlation (r = 0.269; p = 0.0001) was found between hemoglobin level, BMI and waist to hip ratio of e-waste recyclers and women. While a moderate positive correlation (r = 0.453; p < 0.0001) was found between hemoglobin level, waist to hip ratio and body mass index of e-waste recyclers, a weak positive correlation (r = 0.295; p = 0.03) was found among the women in this study (Appendix D). Meanwhile, hemoglobin levels were not correlated with anthropometric measures among e-waste recyclers, women and children in this study.

##### Association between Hemoglobin levels, sociodemographic and anthropometric measures

The unadjusted and adjusted linear regression coefficients are presented in Table 5. Overall, age, daily income, BMI and sex were independently and positively associated with the hemoglobin levels of participants. Generally, age was associated with significant increases in hemoglobin levels (*β* = 0.046; 95% Cl: 0.008, 0.085; p = 0.02) and this was particularly observed among the children (*β* = 0.014; 95% Cl: 0.192, 0.220; p < 0.05). In the unadjusted model, increases in daily income earned was associated with significant increases in hemoglobin levels (*β* = 0.273; 95% CI: 0.163, 0.382; *p* < 0.0001) of all participants. Male e-waste recyclers generally had significantly higher hemoglobin levels (*β* = 2.242; 95% CI: 1.638, 2.846; *p* < 0.0001) than adult females. Similarly, among children, females (*β* = 1.644; 95% CI: −2.994, −0.294; *p* = 0.02) had lower hemoglobin levels compared to males. Increased BMI was positively associated with higher hemoglobin levels in e-waste recyclers (*β* = 0.098; 95% CI: 0.004, 0.193; *p* = 0.04) in the adjusted model. Furthermore, higher level of education was associated with higher hemoglobin levels (*β* = 1.566; 95% CI: 0.435, 2.697; p < 0.01) of children in the adjusted model. Children who consumed food from less than five food groups had significant decreases in hemoglobin levels [*β* = −1.789; 95% Cl: −3.531, −0.047; *p* = 0.04].

## Discussion

Our study assessed the prevalence of anemia and its association with sociodemographic and anthropometric factors among e-waste recyclers, women and children living at Agbogbloshie, an informal e-waste recycling hub in Ghana, particularly known for environmental pollution. The findings indicate a high prevalence of anemia among women and children, with more than 80% of the participants having poor dietary diversity scores

We controlled for menses among women in our design as mentioned in our methods, yet anemia was prevalent among women as well as children in this study. Anemia, defined by low hemoglobin levels is a common condition in resource-poor settings and can result from inadequate dietary intake of iron and other essential nutrients as well as the exposure to toxic environmental substances. In Agbogbloshie, these factors intersect, compounding the risk of anemia among vulnerable populations. The exposure to toxic metals including lead, cadmium and mercury is has consistently been prevalent among e-waste recyclers at Agbogbloshie in at least the last decade [33, 48, 49, 50]. Other authors have highlighted the potential of toxic metals to interfere with iron metabolism and hinder hemoglobin synthesis, thereby exacerbating anemia risk [20, 50, 51]. Previous studies on health risks associated with the Agbogbloshie e-waste site have primarily focused on e-waste recyclers, often neglecting the health impacts on nearby residents, particularly vulnerable groups such as women and children. These populations although not directly involved in recycling activities, are consistently exposed to toxic pollutants due to their proximity to the site. Women and children residing in this area may be exposed to high levels of toxic metals which have been shown to contribute to anemia and other serious health conditions [1, 21, 52, 53, 54, 55]. Compounding the issue, dietary intake in Agbogbloshie is generally poor with limited consumption of iron-rich foods such as leafy greens, iron rich organ meat, eggs, milk and dairy products all of which are crucial for preventing anemia.

The dietary diversity score (DDS) is a useful indicator for assessing overall diet quality, nutrient adequacy, and diet–disease associations [56]. However, some authors have indicated the lack of dietary diversity as a common among resource-poor population from low-and middle-income countries (LMICs), given their diets are largely based on starchy staples, with little or no animal products and few fresh fruits and vegetables [57, 58, 59, 60]. Consistent with our findings, many studies in Ghana [61, 62, 63, 64, 65, 66] and elsewhere [67, 68, 69, 70] have reported poor diet quality among children, as well as adult males and females. The identified reasons for low dietary diversity scores (DDS) include limited women’s empowerment, insufficient educational campaigns on nutrition, inadequate household food insecurity interventions, and socioeconomic challenges. Meanwhile, children who consumed food from fewer than five food groups exhibited significant decreases in hemoglobin level in this study. This may be due to inadequate intake of essential nutrients, such as iron, vitamins, and minerals that are crucial for hemoglobin synthesis [71, 72, 73]; emphasizing the need of a varied diet for nutritional benefits. The poor diet quality (DDS) recorded in our study following the FANTA Project guidelines, highlight a critical dietary gap among e-waste recyclers, women, and children living near e-waste sites like Agbogbloshie in Ghana. These groups showed a limited intake of iron-rich foods, such as dark green leafy vegetables, organ meats rich in iron and legumes, seeds and nuts, which are known to support healthy hemoglobin levels and prevent iron-deficiency anemia. Iron is an essential component of hemoglobin, the protein in red blood cells responsible for oxygen transport, and inadequate iron intake can lead to anemia, reduced immune function, and impaired cognitive development, particularly in children [74, 75]. Women of reproductive age are also susceptible to iron deficiency anemia due to menstrual blood loss and increased iron needs during pregnancy [76], and in low-resource areas, they are less likely to receive adequate iron supplementation or dietary support. Though poorly consumed by study participants, eggs and dairy products are excellent sources of high-quality protein, essential vitamins, and minerals, including vitamin B12, riboflavin, calcium, and phosphorus, all of which play critical roles in growth, bone health, and overall body function [77]. Despite the known benefits of iron-rich foods, their consumption remains low in these populations due to a combination of economic constraints, limited food availability, and lack of dietary knowledge. Dark green leafy vegetables and organ meats, legumes, seeds and nuts and other fruits rich in Vitamin A while locally available, are often not prioritized due to higher costs, dietary preferences, taste for western diets or lack of awareness about their nutritional benefits [78]. Furthermore, a combination of biological vulnerability and contextual factors likely explains the observed higher prevalence of severe acute malnutrition among male children in this study. Consequently, future studies may focus on context-specific assessments to help clarify the underlying causes.

Past findings suggesting an association between anthropometric measures and hemoglobin levels remains varied. We observed an association between BMI and hemoglobin levels among women and e-waste recyclers together. However, we found no association between anthropometric measures-such as height-for-age, weight-for-age and BMI-for-age and hemoglobin levels among children aged 5 to 17 years in this study. Some authors have suggested a potential explanation for the lack of association between hemoglobin and anthropometric measures. They suggest that hemoglobin levels are primarily affected by dietary intake and absorption of iron and other micronutrients, rather than overall growth or body composition [79, 80, 81]. For instance, a child could exhibit adequate growth in terms of height and weight but still have low hemoglobin levels due to insufficient dietary intake of iron-rich foods or poor iron absorption caused by factors like infections, inflammation, or intestinal parasites [82, 83]. This underscores the point that anthropometric measures assess general nutritional status but may not always reflect micronutrient deficiencies. Other authors have also suggested possible reason for the lack of association between hemoglobin levels and anthropometric measures to be the multifactorial nature of hemoglobin regulation. They suggest that hemoglobin levels are influenced by various factors, including dietary diversity, socioeconomic status, parental education, and access to healthcare [82, 84, 85]. In contrast, anthropometric measures are more likely influenced by overall caloric and macronutrient intake, which may not correlate with the specific nutrients required for hemoglobin synthesis [86]. This disconnect could also explain why no association was observed in this study. Notwithstanding these, our finding highlights the importance of using complementary approaches to assess nutritional status in children. While anthropometric measures are valuable for identifying malnutrition, they may not capture hidden hunger–micronutrient deficiencies like iron deficiency anemia that occur even in children with normal growth patterns. This underscores the need for integrating biochemical markers, such as hemoglobin and ferritin levels, into nutritional assessments for a more comprehensive understanding of child health.

The significant association between age and hemoglobin levels among children, similar to past findings [87, 88] may likely be due to several developmental and nutritional factors that evolve with age. Our study highlights an important finding regarding the hemoglobin levels of children aged 12 to 17 years, who did not meet the recommended reference levels for hemoglobin. This gap between recommended and actual hemoglobin levels in adolescents is concerning, as hemoglobin is crucial for oxygen transport, and insufficient levels can lead to anemia, which in turn impacts overall health, development, and cognitive function [89, 90]. The observed difference in hemoglobin levels between males and females, aligns with established physiological patterns and is attributed to several biological, hormonal, and nutritional factors. Females often exhibit lower hemoglobin levels due to menstrual blood loss and lower iron stores, both of which contribute to an increased risk of anemia [91, 92]. Furthermore, our findings suggesting education as a predictor for increasing hemoglobin levels points an important intersection between health literacy and nutrition, which plays a central role in hemoglobin production.

Studies indicate that addressing anemia in populations facing multiple risk factors requires a multi-faceted approach. Consequently, nutritional interventions, such as provision of iron and folic acid supplementation, along with dietary education to improve the intake of locally available iron-rich foods, may help mitigate the risk of anemia among residents in Agbogbloshie. In addition, public health measures aimed at reducing exposure to toxic metals through cleaner and sustainable e-waste management practices could provide long-term health benefits for the community. Biomonitoring of toxic metal exposure and regular health screenings for anemia could provide valuable data to guide these interventions. Here, we provide valuable baseline insights into the prevalence of anemia among e-waste recyclers, women, and children in Agbogbloshie, and its association with sociodemographic and anthropometric factors. The study’s strengths lie in its focus on a high-risk population, the comprehensive analysis of sociodemographic and anthropometric factors, and the recognition of environmental risks. However, its limitations such as the cross-sectional design may not allow for generalizability of findings hence suggests the need for future research using more robust designs to establish causal links.

## Conclusion

In conclusion, the study underscores anemia as a significant public health issue in Agbogbloshie, particularly among women, and children. The high prevalence of anemia was found to be associated with sociodemographic factors such as age, gender, education level, and socioeconomic status. Addressing these underlying factors through targeted interventions such as improving and increasing nutrition and health education, as well as reducing environmental exposures remains crucial to alleviating the burden of anemia in this community. Furthermore, our findings point to the urgent need for policy reforms to improve nutritional and environmental health in vulnerable and toxicant exposed communities like Agbogbloshie.

## Supplementary Material

Supplementary Files

This is a list of supplementary files associated with this preprint. Click to download.

• Appendices.docx

## Figures and Tables

**Table 1:** Anthropometric measurements for Children, E-waste recyclers and Women

Children (aged 5–17 years)					
Measures	Overall Mean ± SD	Male Mean ± SD	Female Mean ± SD	z-score	*p*-value
Weight-for-age	20.66 ± 6.51	21.66 ± 6.46	19.25 ± 6.47	1.276	0.202
Length/ Height-for-age (Stunting)	29.60 ± 4.51	31.14 ± 4.90	27.45 ± 2.81	3.032	0.002**
BMI-for-age	0.47 ± 1.94	0.01 ± 1.43	1.12 ± 2.36	−1.178	0.075
Waist circumference (cm)	65.06 ± 11.17	65.82 ± 8.71	63.99 ± 14.09	0.031	0.975
Hip circumference (cm)	77.53 ± 9.65	77.71 ± 9.31	77.27 ± 10.34	0.283	0.777
Waist-to-hip ratio	0.84 ± 0.04	0.83 ± 0.05	0.85 ± 0.03	−1.910	0.056
MUAC	16.97 ± 2.56	16.83 ± 2.50	17.17 ± 2.66	−0.220	0.826
Indicators Measured	Overall Frequency N(%)	Male n(%)	Female n(%)	χ^2^	p-value
BMI-for-Age Categorization				7.076	0.132
Thinness/ Wasting	5(10.42)	3(10.71)	2(10.00)		
Healthy Weight	26 (54.17)	18 (64.29)	8 (40.00)		
At-risk-of-overweight	6(12.50)	4(14.29)	2(10.00)		
Overweight	8 (16.67)	3(10.71)	5 (25.00)		
Obese	3 (6.25)	0 (0.00)	3(15.00)		
MUAC Classification				3.943	0.139
Normal	4 (8.33)	1 (3.57)	7 (25.00)		
Moderate Acute Malnutrition (MAM)	20 (41.67)	10(35.71)	10 (50.00)		
Severe Acute Malnutrition (SAM)	24 (50.00)	17 (60.71)	7 (25.00)		
Height-for-age				0.499	0.480
Normal	44 (91.67)	25 (89.28)	19 (95.00)		
Moderate Stunting	4 (8.33)	3 (10.71)	1 (5.00)		
Severe Stunting	0 (0.00)	0 (0.00)	(0.00)		
E-waste recyclers and Women (aged 18 to 55 years)					
	Overall Mean ± SD	Recyclers Mean ± SD	Women Mean ± SD	z-score	p-value
Weight (kg)	64.95 ± 12.31	66.56 ± 11.57	61.84 ± 13.17	2.32	0.02*
Height (m)	1.68 ± 0.08	1.71 ± 0.06	1.61 ± 0.07	8.89	< 0.01**
Body Mass Index (BMI) (kg/m^2^)	23.63 ± 6.27	23.02 ± 4.20	24.82 ± 8.95	−1.73	0.09
Waist circumference (cm)	80.57 ± 12.69	79.45 ± 13.33	82.73 ± 11.14	−1.55	0.12
Hip circumference (cm)	96.37 ± 10.22	95.03 ± 8.87	98.96 ± 12.09	−2.33	0.02*
Waist-to-hip ratio	0.85 ± 0.09	0.85 ± 0.10	0.84 ± 0.06	1.13	0.26
Systolic BP	119.37 ± 15.79	123.69 ± 12.42	111.04 ± 18.19	5.15	< 0.01**
Diastolic BP	74.26 ± 10.22	75.38 ± 10.55	72.11 ± 9.27	1.92	0.06
Pulse Pressure	79.72 ± 14.79	76.05 ± 15.43	86.78 ± 10.41	−4.59	< 0.01**

**Table 2:** Dietary diversity scores of e-waste recyclers women and children assessed in 2022

Food Groups	Consumed in the past 48 hours			
	Total	E-waste Recyclers	Women	Children
	*N*(%)	n (%)	n (%)	n (%)
Cereal and Cereal Products	200 (97.55)	104 (100)	48 (90.56)	47 (100)
Dark green leafy vegetables	41 (20.20)	23 (22.12)	17 (33.33)	1 (2.08)
Milk and dairy products	81 (39.90)	41 (39.42)	18 (35.29)	22 (45.83)
Meat and Fish	162 (78.64)	94 (90.38)	33 (61.11)	35 (72.92)
Eggs	88 (43.35)	36 (34.62)	19 (37.25)	33 (68.75)
Legumes, nuts and seeds	135 (66.50)	67 (64.42)	37 (72.55)	31 (64.58)
Fruits and vegetables	197 (95.63)	104 (100)	49 (90.74)	44 (91.67)
Organ meat rich in iron	9 (4.43)	6 (5.77)	3 (5.88)	0 (0)
Other vitamin A rich fruits and vegetables	119 (57.77)	60 (57.69)	34 (62.96)	25 (52.09)
Dietary diversity of participants				
<Five food groups	170 (83.74)	91 (87.50)	39 (76.47)	40 (83.33)
≥Five food groups	33 (16.26)	13 (12.50)	12 (23.53)	8 (16.67)

**Table 3:** Mean Hemoglobin levels among Children, E-waste recyclers and Women

Children		
Anemia Status	Mean Hb (g/dL) Mean ± SD	*p*-value
**Overall** Hb Level (g/dL)	11.68 ± 2.66	
**Hb by Gender**		0.19
Male	12.24 ± 1.22	
Female	10.88 ± 3.78	
**Age categories (years)**		0.10
6–11 years (males and females)	12.07 ± 0.63	
12–14 years (males and females)	10.85 ± 3.34	
15–17 years (males)	11.78 ± 1.03	
15–17 years (females)	10.40 ± 4.65	
**E-waste recyclers and Women**		
**Overall** Hb Level (g/dL)	13.58 ± 2.20	
**Hb by Gender**		
E-waste recyclers	14.65 ± 1.25	< 0.001**
Women	11.60 ± 2.30	
**Age categories (years)**		0.003**
18–30	13.32 ± 2.25	
31–40	14.82 ± 1.54	
41–50	14.80 ± 1.73	
≥ 51	14.2 ± 1.54	

**Table 4:** Prevalence and severity of Anemia among Children, E-waste Recyclers and Women

	Overall Frequency n (%)	Male n (%)	Female n (%)	χ^2^	*p*-value
	**Children (5–17 years )**				
**Anemia Prevalence**		14 (50.00)	8 (44.44)	0.135	0.713
**Severity of Anemia**				2.850	0.241
No Anemia	24 (52.17)	14 (50.00)	10 (55.56)		
Mild Anemia	18 (39.13)	10 (35.71)	8 (44.44)		
Moderate Anemia	4 (8.70)	4 (14.29)	0 (0.00)		
Severe Anemia	0 (0.00)	0 (0.00)	0 (0.00)		
**E-waste Recyclers and Women (18 or more years )**					
**Anemia Prevalence**		9 (9.00)	28 (54.90)	38.47	< 0.001**
**Severity of Anemia**				40.57	< 0.001**
No Anemia	114 (75.50)	91 (91.00)	23 (45.10)		
Mild Anemia	25 (16.56)	8 (8.00)	17 (33.33)		
Moderate Anemia	10 (6.22)	1 (1.00)	9 (17.65)		
Severe Anemia	2 (1.32)	0 (0)	2 (3.92)		

* *p* < 0.05;

** *p* < 0.01;

*** *p* < 0.001.

**Table 5:** Sociodemographic and anthropometric measures associated with hemoglobin levels of e-waste recyclers, women and children at Agbogbloshie, Ghana, 2022

	Hemoglobin levels (g/dL)					
	E-waste recyclers		Women		Children	
	Unadjusted *β* (95% CI)	Adjusted *β* (95% CI)	Unadjusted *β* (95% CI)	Adjusted *β* (95% CI)	Unadjusted *β* (95% CI)	Adjusted *β* (95% CI)
Age	0.042 [0.013, 0.071]**	0.020 [−0.016, 0.056]	0.073 [−0.005, 0.151]	0.018 [−0.067, 0.104]	0.0001[−0.285, 0.256]	−0.360 [−0.654 0.066]*
Daily income	0.068 [−0.037, 0.173]	0.030 [−0.071, 0.131]	0.106 [−0.147, 0.359]	0.047 [−0.208, 0.302]	0.014 [−0.192, 0.220]	−0.036 [−0233, 0.161]
Educational Level	−0.201 [−0.412, 0.010]	−0.173 [−0.376, 0.031]	0.077 [−0.530, 0.684]	0.254 [−0.339, 0.848]	1.254 [0.100, 2.408]*	1.566 [0.435, 2.697]**
BMI-for-age	-	-	-	-	−0.071 [−0.479, 0.336]	−0.018 [−0.430, 0.394]
BMI	0.098 [0.042, 0.154]**	0.098 [0.004, 0.193]*	0.066 [−0.003, 0.135]	0.025 [−0.047, 0.097]	-	-
DDS	-	-	-	-	-	-
< 5 food groups	1	1	1	1	−1.180 [−3.249, 0.889]	−1.789 [−3.531, −0.047]*
≥ 5 food groups	−0.166 [−0.934, 0.602]	−0.055 [−0.799, 0.689]	−0.027 [−1.760, 1.706]	0.2636365 [−1.377, 1.904]	1	1
Waist -to- hip ratio	3.068 [−0.167, 6.303]	-	8.055 [−3.111, 19.222]	-	−2.479 [−20.470, 15.511]	0.724 [15.844, 17.293]
Sex	-		-	-		
Female	-	-	-	-	−1.361[−2.896, 0.173]	−1.644 [−2.994, −0.294]*
Male	-	-	-	-	1	1
Height for age	-	-	-	-	−0.158 [−0.628, 0.312]	-
MUAC	-	-	-	-	−0.023 [−0.332, 0.286]	-
MUAC for age	-	-	-	-	0.366 [−0.873, 1.606]	-

Variables with *p* ≥ 0.25 in the bivariable (unadjusted) regression were excluded from the multivariable (adjusted) regression;

* *p* < 0.05;

** *p* < 0.01;

*** *p* < 0.001.

## Data Availability

Data that support the findings of this study are available from the corresponding author upon reasonable request.
